# Eosinophilic Ascites: An Infrequent Presentation of Eosinophilic Gastroenteritis

**DOI:** 10.7759/cureus.24303

**Published:** 2022-04-20

**Authors:** Mafalda Sequeira, Daniela Cruz, Francisca Abecasis, Henrique Santos, Francisca Delerue

**Affiliations:** 1 Internal Medicine, Hospital Garcia de Orta, Almada, PRT

**Keywords:** abdominal pain, ascites, eosinophilic mucosal infiltration, benign inflammatory disorders, eosinophilic gastroenteritis diagnosis

## Abstract

Eosinophilic gastroenteritis (EGE) is an unusual and benign inflammatory disorder that mainly affects the digestive tract. Its main symptoms are cramp-like abdominal pain, nausea, vomiting, diarrhea, gastrointestinal bleeding, and weight loss. Laboratory results show peripheral eosinophilia. This disease generally affects patients with a personal history of atopy and drug or food intolerance. The etiology remains unknown, the diagnosis is challenging, and the treatment depends on the severity of the disease and can range from supportive therapy to corticosteroid therapy.

We report a case of a 24-year-old female known to have a history of iron deficiency anemia who was brought to the emergency department with an intense colicky abdominal pain, fatigue, diarrhea, and vomit right after a mild coronavirus disease 2019 (COVID-19) infection. The clinical investigation revealed moderate ascites identified in abdominal computed tomography (CT) scan, peripheral blood eosinophil count, and elevation of inflammatory parameters. An ultrasound-guided diagnostic paracentesis was performed, showing ascitic fluid with a clear predominance of eosinophils (57%). To confirm the diagnosis of EGE, an upper digestive endoscopy (UDE) was performed. The biopsies of the esophagus and gastric body revealed polymorphonuclear eosinophils and colonic mucosal biopsies revealed eosinophils (20 eosinophils per 10 fields). After reviewing the clinical history, we concluded that the patient was taking iron supplements due to her iron deficiency anemia, whose onset coincided with the symptoms presented. Exploring the clinical history a little more, the patient mentioned that in the past, she already had some intolerance to oral iron supplements, manifested by gastrointestinal symptoms, although milder. Approximately three weeks after suspending the supplements, we have seen an analytical improvement that was accompanied by clinical improvement. The patient was discharged with the resolution of abdominal pain.

## Introduction

First described by Kaijser in 1937, eosinophilic gastroenteritis (EGE) is an atypical condition involving eosinophilic infiltration of the intestinal wall [1]. The occurrence of EGE is estimated to be about 1-30/100,000, and it is invariably a diagnostic challenge for clinicians [2]. The illness is classified into three subtypes (mucosal, muscular, and serosal) in consonance with Klein’s classification, and its manifestations vary with the involved digestive tract segment. The following three criteria are needed for diagnosis: suspicious clinical symptoms, histologic evidence of eosinophilic infiltration in the bowel, and exclusion of other pathologies.

Owing to the non-specific character of the symptoms of EGE, the clinicians hardly think of EGE, except if cramp-like abdominal pain, nausea, vomiting, diarrhea, gastrointestinal bleeding, and weight loss, which are the most common symptoms, are refractory or elevated eosinophils are present on peripheral blood [3].

Allergic diseases, including asthma, rhinitis, eczema, and drug or food intolerances, are found in 45-63% of the reported EGE cases; moreover, 64% of the reported cases include a family history of atopic diseases [4]. The authors also point out that, although the diagnostic criteria (Klein’s classification) include complementary diagnostic tests, EGE is a disease with a fundamentally clinical diagnosis.

Treatment depends on the severity of the disease and can range from supportive therapy to corticosteroid therapy; for this reason, the infection with *Strongyloides stercoralis* should be excluded because this infection can become life-threatening if systemic immunosuppression is necessary. Early recognition of this illness is essential to improve our diagnostic and therapeutic abilities [3].

We introduce a case of a young woman with a challenging diagnosis of EGE, post mild coronavirus disease 2019 (COVID-19) infection.

## Case presentation

A 24-year-old female with a history of iron deficiency anemia due to menorrhagia presented to the emergency department with complaints of progressively worsening fatigue, bloating, and abdominal pain a few days after ending isolation due to mild COVID-9 (fatigue, myalgias, and cough). The patient described colicky abdominal pain in the lower quadrants, with an intensity of 8/10, which was worse at night, and with no correlation to meals. The patient referred to the use of oral analgesics and lateral decubitus as relieving factors. Cough, abdominal wall contraction, and trembling movements were worsening factors and indicative of possible peritoneal irritation. Concomitant to the onset of abdominal pain, the patient also had food vomiting and posteriorly bilious vomiting, lasting for about four days and resolving with antiemetic therapy.

**Figure 1:**
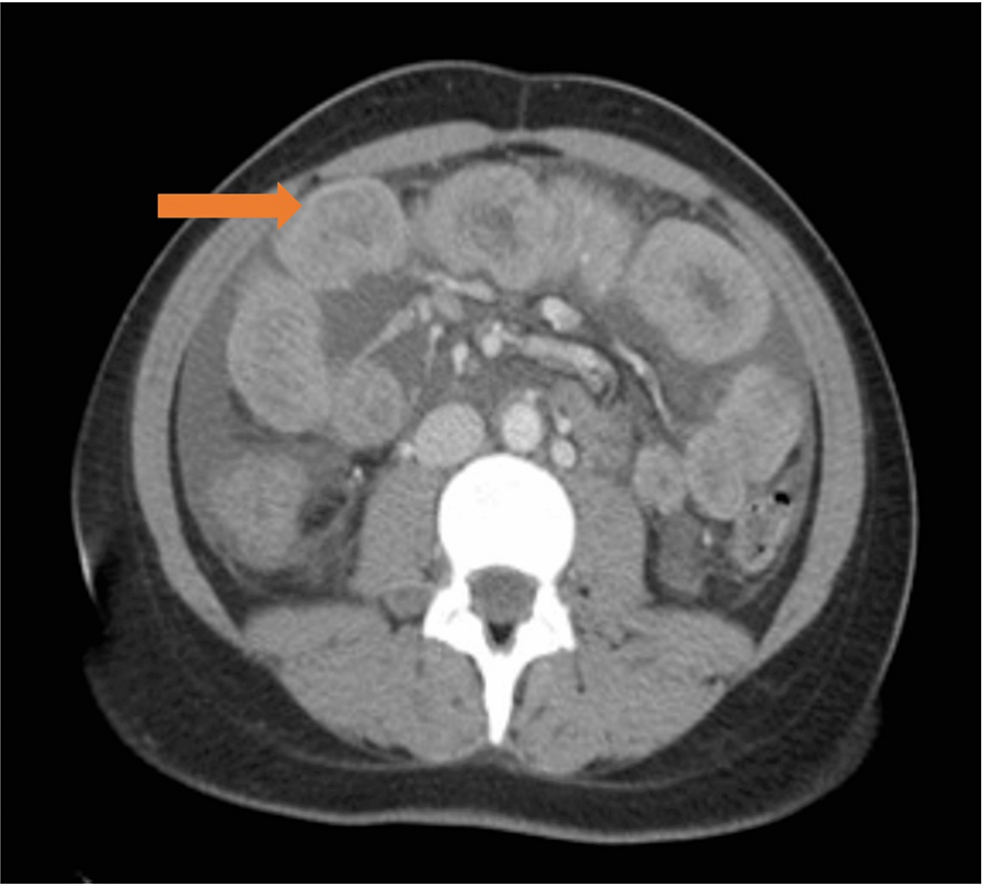
CT scan (orange arrow) highlighting parietal thickening involving duodenum and proximal jejunum of probable inflammatory nature.

On physical examination, the patient was hemodynamically stable, apyretic, with a globose, distended, and diffusely painful abdomen on deep palpation, without peritoneal reaction. Blood analyses showed mild leukocytosis with neutrophilia and slightly increased C-reactive protein (CRP). An abdominal computed tomography (CT) scan was performed, which revealed moderate ascites and stratified parietal thickening involving the stomach, duodenum, and proximal jejunum of probable inflammatory nature (Figure 1). For this reason and considering that the condition could be correlated with COVID-19 infection, the patient was hospitalized in the infectious diseases department with the suspicion of infectious colitis and fulfilled seven days of antibiotic therapy. About six days after discharge, the patient developed diarrhea (stool frequency > five per day), without blood or mucus, associated with worsening of the previously described abdominal pain, and returned to the emergency department.

**Figure 2:**
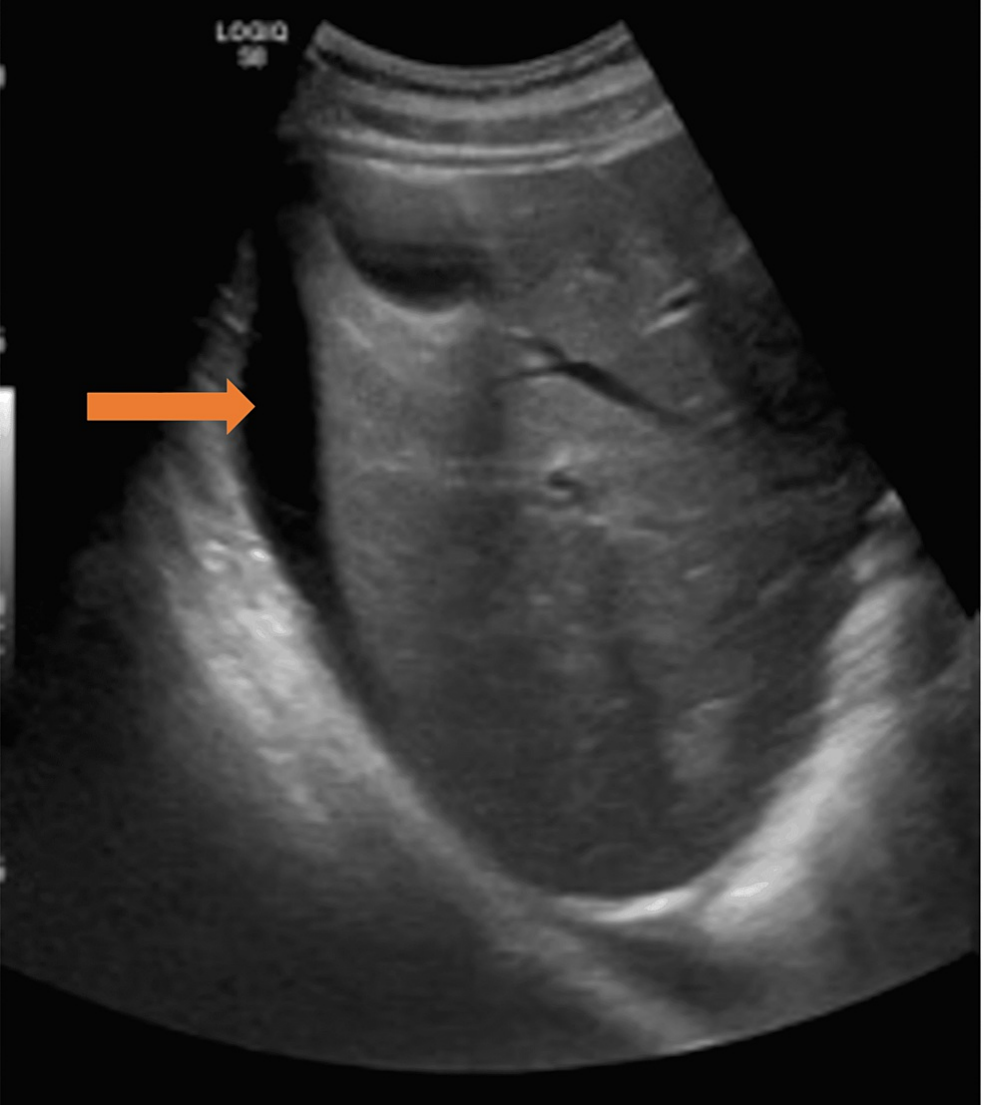
Abdominal ultrasound (orange arrow) highlighting moderate ascites in the right upper abdominal quadrant.

**Figure 3:**
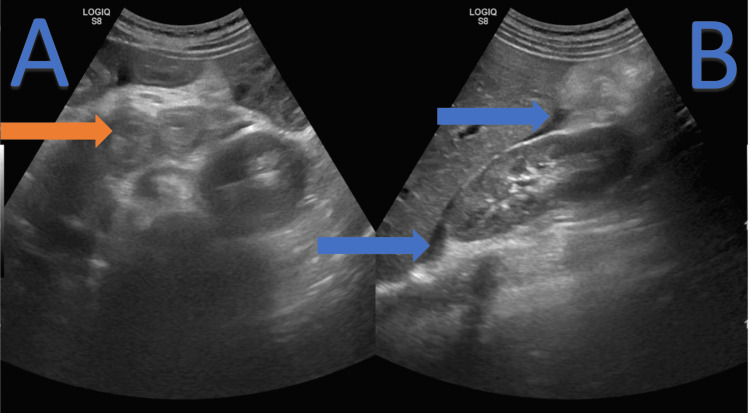
(A) Abdominal ultrasound (orange arrow) highlighting stratified parietal thickening involving the duodenum. (B) Blue arrows highlighting moderate ascites in Morrison space.

The new evaluation revealed increased eosinophils (17%; 1.59 10^9/L). An abdominal ultrasound was performed, which proved to be globally superimposed to the previous abdominal CT scan and additionally revealed a slight right pleural effusion (Figures 2, 3).

The patient was admitted to the internal medicine department for further investigation. An ultrasound-guided paracentesis was performed, and ascitic fluid revealed 809 cells/uL with a clear predominance of eosinophils (57%), with no other relevant alterations; the bacteriological and mycobacteriological exams were negative. Considering the clinical findings, peripheral eosinophilia, and the changes described in the ascitic fluid, the diagnostic hypothesis of EGE was raised. Concerning that EGE is a diagnosis of exclusion, the diagnostic investigation progressed, and the clinical history was enquired in more detail.

The patient denied recent travels and contact with unvaccinated animals, but reported eating sushi a few days before the onset of symptoms; she was also taking a vitamin supplement with iron in its composition. It was mentioned that in the past, due to iron deficiency anemia, she took iron supplements and had an intolerance reaction, characterized by abdominal pain, diarrhea, and malaise.

Blood electrophoresis revealed an increased total immunoglobulin E (IgE) level of 163 U/mL (0-120), with the remaining measured immunoglobulins (IgM, IgG, IgA) within the reference values.

Blood cultures, stool cultures, and *Clostridium difficile* toxin were all negative. Serology for *Strongyloides stercoralis*, schistosomiasis, anisakiasis, trichinellosis visceral migrating larvae, and hookworm was negative. The patient was negative for HIV-1 and HIV-2 and cytomegalovirus (IgM). The autoimmune study was also negative.

To confirm the diagnosis of EGE, an upper digestive endoscopy (UDE) was performed, which revealed diffuse hyperemia of the esophagus, gastric mucosa, and antrum. Esophagus and gastric body biopsies revealed polymorphonuclear eosinophils in a probable context of active chronic inflammation, without histomorphological criteria for eosinophilic gastritis/esophagitis, and colonic mucosal biopsies revealed eosinophils, with areas of 20 eosinophils per 10 fields. It should be noted that the endoscopic exams were performed in a phase of clinical and analytical improvement, which may explain the absence of histomorphological criteria. During hospitalization, there was an initial worsening of the eosinophil count (2.51 10^9/L; 25.1%), with subsequent improvement with resolution of the eosinophilia. Analytical improvement was accompanied by clinical improvement, and the patient was discharged with the resolution of the abdominal pain and indication to avoid iron-containing supplements. Approximately one month after discharge, the patient performed a new abdominal CT scan, which documented resolution of the ascites and improvement of the antropyloric, duodenal, and jejunal parietal thickening.

## Discussion

EGE can affect the entire digestive tract, from the esophagus to the rectum, and its clinical presentation depends on the location of eosinophilic infiltration [5]. Diarrhea, abdominal pain, weight loss, anemia, nausea, and vomiting are frequent symptoms in adults and nearly 80% of patients have a chronic evolution [6].

All patients with abdominal refractory symptoms, especially those with a strong personal history of atopy, peripheral blood eosinophilia, or history of EGE, must make a clinical evaluation for EGE. Gastrointestinal symptoms and demonstration of gastrointestinal eosinophilia on tissue biopsy must be present for diagnosis, as well as the exclusion of other etiologies [7].

The etiology remains unknown, even if some studies show that patients with EGE have increased secretion of interleukin (IL)-4 and IL-5 mediated by peripheral blood T cells.

All layers of the gastrointestinal tract can be involved, so patients with muscularis subtype, serosal subtype, or both may have endoscopic biopsies with no alterations. It is frequent for the endoscopic appearance of the gastrointestinal tract to be normal, making the diagnosis dependent on the knowledge of the physician who evaluates the biopsy samples [8].

Patients may have multiple symptoms based on the portion of the gastrointestinal tract involved. The mucosal form is the more common presentation (occurs in about 25-100% of cases) and usually presents as abdominal pain, nausea, vomiting, dyspepsia, diarrhea, malabsorption, gastrointestinal hemorrhage, protein-losing enteropathy, and weight loss. The muscularis form, which occurs in about 10-60% of cases, generally attends with obstructive symptoms due to pyloric stenosis and gastric obstruction. The subserosal form, which is less common, usually presents with significant bloating, exudative ascites, and a considerably larger number of peripheral eosinophilia than other forms [9].

Evaluation for intestinal parasites through examination of stool samples, intestinal aspirates obtained during colonoscopy, or specific blood antibody titers should be performed. The infection with *Strongyloides stercoralis* should be excluded because this infection can become life-threatening if systemic immunosuppression is necessary [10].

The severity of symptoms influences the kind of treatment for each patient. Patients with mild symptoms are managed with symptomatic therapy and a careful search of food allergens or medications. Most patients have moderate to severe symptoms and corticosteroids are the base of their therapy [11].

We present a case of a patient with abdominal refractory symptoms, with a personal history of atopy to iron supplements, peripheral blood eosinophilia, and eosinophilic ascites. The histomorphological criteria do not fit EGE diagnostic criteria; however, we cannot fail to mention that the colonoscopy was performed in a phase of the clinical and analytical improvement of the patient, which could explain the absence of findings. We always have to think about other differential diagnoses, such as infectious diseases, that should always be excluded before any therapeutic institution. As we can see with our patient, the avoidance of the allergen (in this case, the iron supplements) was enough to resolve the symptoms. Therefore, we emphasize the importance of collecting a good clinical and pharmacological history.

## Conclusions

The authors present a case of eosinophilic ascites, a rare presentation of EGE. This case highlights the fact that EGE is an uncommon entity and it should be kept in mind in patients with unexplained ascites. It is frequent for the endoscopic appearance of the gastrointestinal tract to be normal since all layers of the gastrointestinal tract may be involved, which makes diagnosis challenging. Recognizing the symptoms and analytical changes enables early diagnosis and appropriate treatment, which are essential for the successful management of this disease.
